# Ill Effects of Quinine

**Published:** 1857-06

**Authors:** 


*Ill Effects of Quinine.*—Dr. Greenwald, of Cincinnati, reports a case in the Western Lancet, in which decided and persistent hemiplegia was produced by the use of quinine. About twenty grains was administered in three doses combined with other remedies. The hemiplegia was preceded by ringing in the ears and a disposition to sleep. This case, with others on record, shows that the administration of quinine is not, under all circumstances, unattended with danger.—*Ibid.*

